# Paraguay’s approach to biotechnology governance: a comprehensive guide

**DOI:** 10.3389/fbioe.2024.1373473

**Published:** 2024-03-27

**Authors:** Nidia Benítez Candia, María Gabriela Ulke Mayans, Pablo Hernán Sotelo, Eva Nara Pereira, Andrea Alejandra Arrúa Alvarenga, Danilo Fernández Ríos

**Affiliations:** ^1^ Departamento de Biotecnología, Facultad de Ciencias Exactas y Naturales, Universidad Nacional de Asunción, San Lorenzo, Paraguay; ^2^ Departamento de Relaciones Humanas, Ministerio de Salud Pública y Bienestar Social, Asunción, Paraguay; ^3^ Departamento de Biotecnología, Facultad de Ciencias Químicas, Universidad Nacional de Asunción, San Lorenzo, Paraguay; ^4^ Departamento de Biología Molecular y Biotecnología, Instituto de Investigaciones en Ciencias de la Salud, Universidad Nacional de Asunción, San Lorenzo, Paraguay; ^5^ Mycology Investigation and Safety Team, Centro Multidisciplinario de Investigaciones Tecnológicas, Universidad Nacional de Asunción, San Lorenzo, Paraguay

**Keywords:** Mercosur, genetically modified organisms, recombinant DNA, biologics, vaccines

## Abstract

This study analyzes Paraguay’s biotechnology regulatory framework and its alignment with international standards amid biotechnological advancements. It also identifies areas of improvement for enhancing framework effectiveness. Through this work, we aim to provide a resource for policymakers, stakeholders, and researchers navigating Paraguay’s biotechnology regulation.

## 1 Introduction

The regulation of biotechnology products is a complex process involving various institutions with different protection goals to ensure their safety and efficacy (Wolt and Wolf, 2018). Therefore, it is essential to have a sound regulatory framework to monitor and evaluate biotechnology developments and their applications (Xue and Shang, 2022).

This study presents a compilation of Paraguay’s current regulatory framework for biotechnology focusing on recombinant DNA-derived products, vaccines, and biopharmaceuticals. It examines the country’s efforts to create a regulatory environment that aligns with international standards while accommodating rapid biotechnological advancements. Even though any attempt to summarize and analyze regulations runs the risk of being incomplete and outdated, we are confident that it can serve as a resource for policymakers, researchers, and other stakeholders working to understand and navigate this complex regulatory landscape. We aim to provide an analysis of this regulatory framework, focusing on the roles and responsibilities of the various government bodies involved (Table 1). Finally, we aim to highlight potential areas for improvement and collaboration among stakeholders.

**TABLE 1:** Paraguayan government bodies involved in the regulation of biotechnology and their responsibilities relevant to the topic.

Institution	Responsibilities
Ministry of Agriculture and Livestock (MAG)	• Authorize regulated trials, pre-commercial release, commercial release, and other proposed uses of GMOs to be incorporated into agricultural and forestry production based on the opinion issued by the National Agricultural and Forest Biosafety Commission (CONBIO) (Presidencia de la República del Paraguay, 2012a).
National Service for Animal Quality and Health (SENACSA)	• Supervise biosafety conditions of the introduction of GMOs approved by MAG within the scope of its authority (Presidencia de la República del Paraguay, 2012a).
	• Evaluate GMO feed safety according to intended use (Presidencia de la República del Paraguay, 2012a).
	• Establish requirements for the registration, licensing, manufacturing conditions, commercialization, supervision and prohibition of biological, chemical, pharmacological and food products and supplies for veterinary use (República del Paraguay, 2004a, secs. 8h, 8n).
	• Establish animal health and quality requirements for the import and export of animals, genetic material, products, byproducts of animal origin, products and supplies for veterinary use (República del Paraguay, 2004a, secs. 8h, 8n).
National Service for Plant and Seed Quality and Health (SENAVE)	• Supervise biosafety conditions of the introduction of GMOs approved by MAG within the scope of its authority (Presidencia de la República del Paraguay, 2012a).
	• Ensure the identity and quality of seeds, and protect the right of creators of new cultivars (República del Paraguay, 2004b, secs. 6e, 9e).
	• Develop, and execute programs to improve the phytosanitary quality of products and byproducts of plant origin derived from the use of biotechnology (República del Paraguay, 2004b, secs. 6e, 9e).
National Forestry Institute (INFONA)	• Supervise biosafety conditions of the introduction of GMOs approved by MAG within the scope of its authority (Presidencia de la República del Paraguay, 2012a).
Paraguayan Institute of Agricultural Technology (IPTA)	• Advise the Ministry of Agriculture and Livestock and other institutions in the formulation and execution of agricultural policy in its sphere of action (República del Paraguay, 2010).
	• Cooperate technically with governmental and non-governmental institutions for research, dissemination and transfer of agricultural technology.
Ministry of Public Health and Social Welfare (MSPyBS)	• Regulate the manufacture, import, distribution, and sale of medicines, food, drugs, chemical products, biological and radioactive products, reagents, and all products for use and application in human medicine in accordance with current legislation (Presidencia de la República del Paraguay, 1998a).
	• Regulate, control, supervise and license activities potentially polluting the environment, in coordination with institutions with responsibility in the environmental sector (Presidencia de la República del Paraguay, 1998a).
National Institute of Food and Nutrition (INAN) of the Ministry of Public Health and Social Welfare	• Supervise the biosafety conditions of the introduction of GMOs approved by MAG within the scope of its authority (Presidencia de la República del Paraguay, 2012a).
	• Evaluate GMO food suitability according to the intended use (Presidencia de la República del Paraguay, 2012a).
National Directorate of Sanitary Surveillance (DINAVISA)	• Regulate, control and supervise health products such as drugs for human use, chemical products, reagents, and all other products for use and application in human medicine (República del Paraguay, 2021).
Ministry of Environment and Sustainable Development (MADES)	• Supervise biosafety conditions of the introduction of GMOs approved by MAG within the scope of its authority (República del Paraguay, 2018).

## 2 International treaties

Paraguay has signed several international treaties related to modern biotechnology which emphasize the conservation of biological diversity, the sustainable use of resources, the equitable sharing of the benefits of genetic resources and the safe handling of genetically modified organisms (GMOs). They also address intellectual property rights and plant variety protection (Table 2).

**TABLE 2:** International treaties relevant to modern biotechnology signed by Paraguay.

Treaty	Key objectives/Provisions	Incorporated to local regulatory framework through
United Nations Convention on Biological Diversity	Conservation of biological diversity; sustainable use of components; fair and equitable sharing of benefits from genetic resources; appropriate access to resources and transfer of technologies; management and control of risks associated with living modified organisms (LMOs)^a^	Law No. 253/1993 (República del Paraguay, 1993)
Paris Convention for the Protection of Industrial Property	Broad protection of industrial property, including agricultural and extractive industries and all manufactured or natural products	Law No. 300/1994 (República del Paraguay, 1994a)
Agreement on Trade-Related Aspects of Intellectual Property Rights (TRIPS)	Exclusion of patentability for plants and animals other than microorganisms; protection of plant varieties either by patents or an effective sui generis system or a combination thereof	Law No. 444/1994 (República del Paraguay, 1994b)
International Convention for the Protection of New Varieties of Plants (1978 version)	Recognition and protection of the rights of breeders of new plant varieties or their successors; provision of protection through a special title or patent, with only one protection form allowed per botanical genus or species	Law No. 988/1996 (República del Paraguay, 1996)
Cartagena Protocol on Biosafety	Ensuring protection in the safe transfer, handling, and use of LMOs resulting from modern biotechnology; focus on transboundary movements and possible adverse effects on biological diversity and human health	Law No. 2309/2003 (República del Paraguay, 2003)
International Treaty on Plant Genetic Resources for Food and Agriculture	Conservation and sustainable use of plant genetic resources for food and agriculture; fair and equitable sharing of benefits arising from their use; sustainable agriculture and food security in harmony with the Convention on Biological Diversity	Law No. 3194/2007 (República del Paraguay, 2007a)

^a^ Definitions of the Cartagena Protocol on Biosafety to the Convention on Biological Diversity (Secretariat of the Convention on Biological Diversity, 2000).

LMO: any living organism that possesses a novel combination of genetic material obtained through the use of modern biotechnology.

Living organism: any biological entity capable of transferring or replicating genetic material, including sterile organisms, viruses and viroids.

Modern biotechnology: the application of *in vitro* nucleic acid techniques, including recombinant deoxyribonucleic acid (DNA) and direct injection of nucleic acid into cells or organelles, or fusion of cells beyond the taxonomic family, that overcome natural physiological reproductive or recombination barriers and that are not techniques used in traditional breeding and selection.

For the purposes of this study, the terms LMO, and GMO, will be considered equivalent and referred to as “GMO” in the main document, in accordance with the prevailing use in the literature.

**FIGURE 1:**
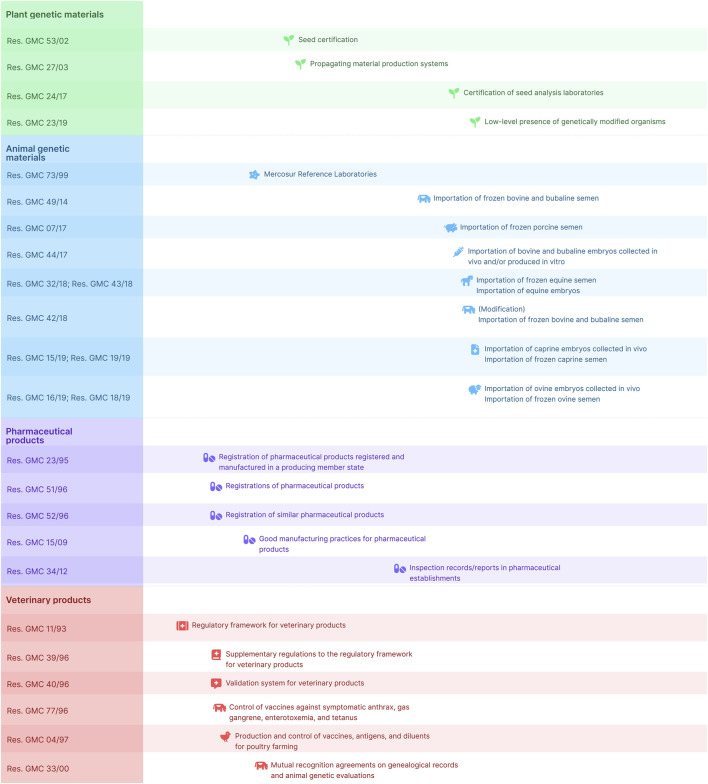
Chronological synthesis of current Mercosur Resolutions. The figure depicts the timeframe and extent of resolutions adopted by the Mercosur Common Market Group concerning biotechnology, offering an overview of key regulatory achievements in the region.

In South America, the Southern Common Market (Mercosur) has been actively developing and implementing regulations for biotechnology products (Figure 1). Mercosur countries recognize the importance of cooperation and harmonization in biotechnology regulation which aim to establish consistent regulations across member states and facilitate trade, while maintaining safety and effectiveness standards (Dellepiane and Pagliusi, 2019). For each of the following sections, we have included Mercosur regulations that address these efforts.

Mercosur Common Market Group (GMC) Resolutions have the goal of setting standards for the harmonization of regulations within the group, namely, once a GMC Resolution is incorporated into a country’s regulatory framework, said country must adjust its internal regulation to fit the standards set by that Resolution, and thus facilitate mutual recognition. To that end, GMC has also passed Resolutions on the procedures to elaborate, revise and revoke technical regulations (Mercosur GMC, 2017), and for mutual recognition of control systems (Mercosur GMC, 1998).

Mercosur’s GMC has long been incorporating Codex Alimentarius guidelines for the harmonization of food safety standards (FAO, 1995; De F Toledo, 2014). However, as of the date of submission of this paper, no Codex guidelines referring specifically to products of biotechnology have been incorporated into the Mercosur framework, and thus the use of said guidelines is left up to each member state.

## 3 Crop biotechnology

**FIGURE 2:**
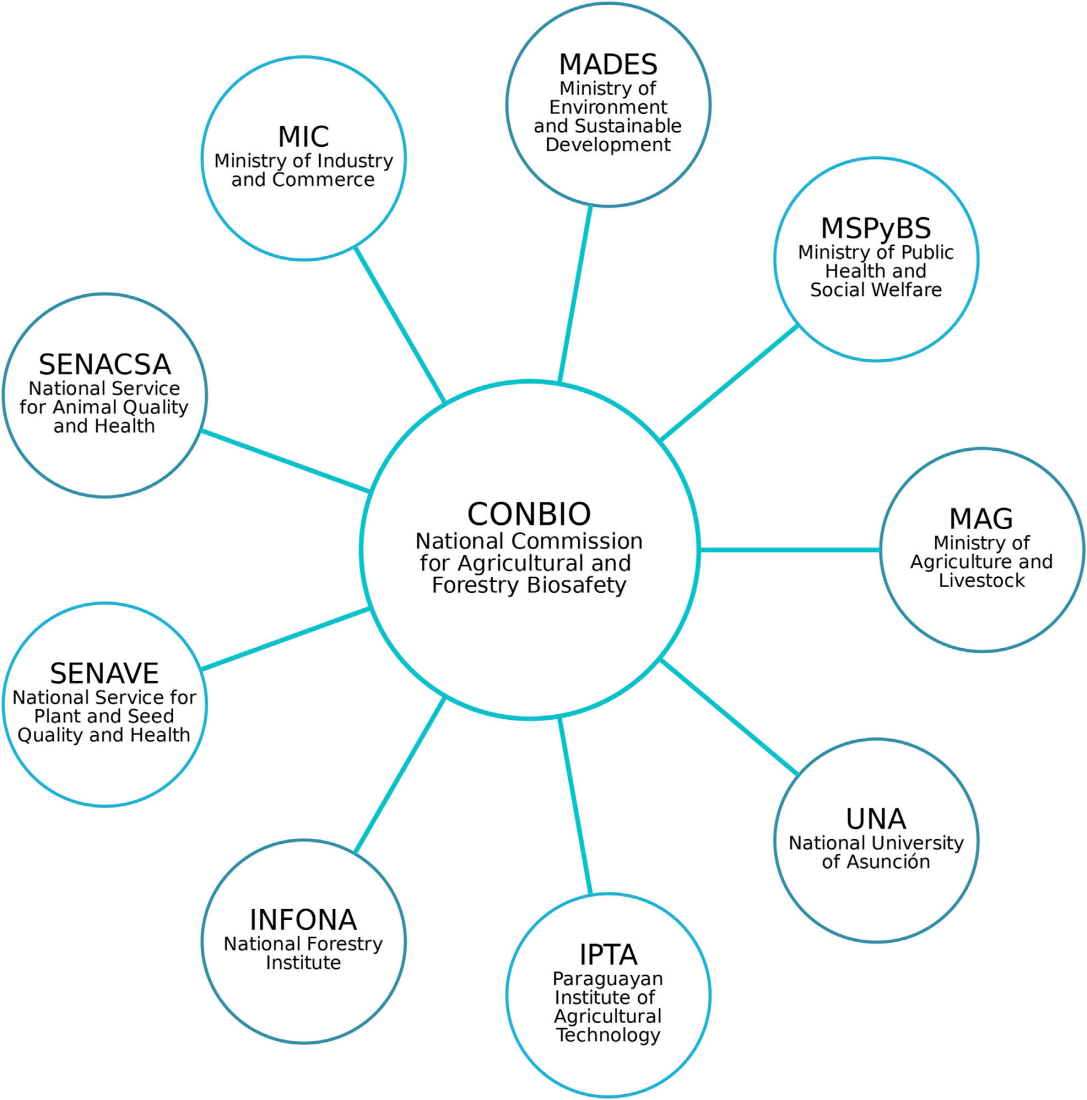
Composition of Paraguay’s National Commission for Agricultural and Forestry Biosafety (Presidencia de la República del Paraguay, 2012a).

MAG plays a central role in authorizing regulated trials, pre-commercial releases, commercial releases, and other proposed uses of genetically modified (GM) crops based on the opinion issued by CONBIO (Figure 2).

Submissions for approval of both regulated field trials and commercial release of plant GM events are received by MAG, and derived for analysis to CONBIO. MAG Resolution No. 27/2015 (MAG, 2015) contains both Form 1, for submission of regulated field trial applications; and Form 2, for submission of commercial release applications for GM crops (Fernandez Rios et al., 2018). Different treatments are established for events submitted for approval, based on their characteristics (Benitez Candia et al., 2020; Fernandez Rios et al., 2024).

In the case of the differentiated procedure established for events previously approved by sound and experienced regulatory systems (MAG, 2019a; MAG, 2019b), it is worth noting that Paraguay has not established a formal definition of an “experienced” regulatory system. However, the country recognizes that such a system employs scientifically sound methodologies for risk assessments to evaluate the safety of GMOs as food and feed and to assess their potential environmental impact. These systems are typically accompanied by several key features (McHughen, 2016), such as regulations that are commensurate with the level of risk and are based on a comparative risk analysis; the development of a rational risk hypothesis; the requirement of scientifically valid data of sufficient quality and quantity to inform on relative safety; and a clear distinction between scientific, political, ethical, and economic considerations.

The agri-food regulatory system is structured around a problem formulation approach, where protection goals are identified and plausible risk hypotheses are formulated. These hypotheses are tested with robust testing methodology. Such tests are conducted either in the laboratory or in confined field trials and follow a tiered-testing approach. For example, for non-target organisms (NTOs) the process starts with highly conservative early-tier tests in the laboratory and progresses to more complex, higher-tier experiments when necessary. Risk assessors must select data that can evaluate potential effects by translating policies and protection goals into risk assessment operational goals (Garcia-Alonso and Raybould, 2014; Wach et al., 2016). Experience has taught local regulators that most countries where GM crops have already been approved have protection goals that apply to a common set of valued ecological functions. One of the key elements that enabled Paraguay to develop a differentiated procedure for GM crops already approved in third countries was the utility of early tier tests using surrogate species for predicting field effects (Wach et al., 2016). The choice of appropriate surrogates for the assessment of NTOs is influenced by scientific factors, such as understanding the mode of action and spectrum of activity, and protection goals based on ecosystem services, including biological control, pollination, and decomposition of organic matter. Furthermore, the Paraguay agri-food regulatory system considers that the results from the use of surrogate species in tiered testing are transportable, as such testing can be replicated in other laboratories (Wach et al., 2016).

When Paraguay updated the regulatory framework for GM crops in 2015, it incorporated the concept of data transportability, which became a useful tool for avoiding redundant confined field trials (CFT). Through collaboration with the regulatory agency from Argentina, risk assessors learned that CFTs’ site selection with a focus on the diversity of environments tested within the production zone of the crop of interest is a key element. At the same time, the appropriate methodology and agronomic management of the studies and the measured endpoints are relevant (Vesprini et al., 2020; Vesprini et al., 2022). If these conditions are met, and if through the application of problem formulation there were no risk hypotheses related to Paraguay’s agro-climatic conditions, not only the data (as informative studies), but also the conclusions of the CFT are transportable, and applicable to risk assessments for Paraguay, without the need to generate additional CFT data.

If a relevant risk hypothesis related to Paraguay’s agro-climatic conditions is formulated, risk assessors scientifically assess the need (or not) for a new specific confined field trial that provides necessary unavailable data and answers that specific risk hypothesis. If a new confined field trial is required, risk assessors need to identify putative agro-climatic zones (Melnick et al., 2023) where this specific confined field trial could be conducted (e.g., where the production of that crop is common, and understanding the importance of any particular agro-climate for the production of a specific crop). This contributes to providing only relevant data that answer a specific risk hypothesis that persists even when all available data are analyzed.

Since 2014, SENAVE has maintained a registry of companies operating with regulated GMOs in the agricultural sector. To apply for registration, companies must fill Forms DBA-01 and DBA-02 (SENAVE, 2014b), whereby they declare relevant data about their legal representatives, technical advisors, and location where the companies operate. SENAVE has also approved a procedure for the risk management of activities with regulated GMOs in the agricultural sector, through Resolution SENAVE No. 283/2014 (SENAVE, 2014a) (Supplementary Table 1). Meanwhile, a Mercosur GMC Resolution has been incorporated into Paraguay’s regulatory framework which approves a mechanism to reduce the low-level presence of unapproved GMOs (Mercosur GMC, 2019; Presidencia de la República del Paraguay, 2021).

## 4 Genetically modified microorganisms

The regulation of genetically modified microorganisms (GMMs) in the agri-food system is governed by the same norm as crop biotechnology and subject to analysis by CONBIO, which includes conducting case-by-case risk assessments of activities involving GMMs and identifying potential environmental risks or safety concerns to human and/or animal health resulting from the use of GMMs and their byproducts (Presidencia de la República del Paraguay, 2012a). However, the differentiated mechanism for events previously assessed in third countries is not currently applicable. Paraguay has approved the use of several GM yeasts for ethanol production (OECD, 2023). Because yeast-derived products and distiller’s dried grains with solubles (DDGS) can be used as animal feed, a CONBIO safety assessment is required.

## 5 Animal products of biotechnology

GM animals have a wide range of applications, from laboratory research to agriculture and public health. The regulation varies depending on these applications. In the context of the agri-food system, an assessment by CONBIO is necessary, and genetically modified animals are regulated according to the same standards as crop biotechnology.

Paraguay has recently granted the first commercial release of a GM insect, *Spodoptera frugiperda*, containing a self-limiting gene which allows for the production of male-only insects (MAG, 2024). Once released into the environment, these modified males will seek out and mate with wild females. The self-limiting gene will be transmitted to offspring, preventing female offspring from reaching maturity and reproducing. By continuously releasing GM males in a specific area, there will be a decrease in the number of wild females and consequently a reduction in the overall population of these insects (Reavey et al., 2022).

For applications related to public health, an evaluation by the MSPyBS may be required in some cases. However, there are currently no established guidelines outlining the assessment process in the public health context.

One particular case is that of synthetic beef (and plant-based meat substitutes). The use of the word “meat” (carne) is regulated by Law No. 6916/2022 (República del Paraguay, 2022), and is reserved for the edible muscular part of animals slaughtered and declared fit for human consumption by the official veterinary inspection, consisting of the soft tissues surrounding the skeleton, including their fat covering, tendons, vessels, nerves, aponeurosis, the skin of swine and poultry (except that of the order Struthioniformes) and all those tissues not separated during the slaughter operation. The diaphragm is also considered meat. At the time of submission of this paper, a draft bill is being studied in Congress to ban synthetic meat altogether (Franco Alfaro, 2023). These laws were promoted by the agribusiness sector as a way to combat market competition for conventional meat, claiming that by calling alternatives ‘meat’, competitors are fooling consumers (La Nacion, 2022). France has a similar policy against using meat-related terms for plant-based products (Carreno, 2022). However, research suggests that labeling these products as “meat” does not inherently cause confusion (Gleckel, 2020; Profeta et al., 2021; Tosun et al., 2021).

Several Mercosur GMC Resolutions have been integrated into the Paraguayan regulatory framework, focusing on animal health requirements for importing various types of animal genetic materials. This includes frozen semen and embryos from bovines, bubalines, porcines, equines, caprines, and ovines (Supplementary Table 2).

## 6 Biologics for human use

The registry of biologics for human use^1^ is made by DINAVISA, following public health policies from MSPyBS and is regulated by the Presidential Decree No. 6611/2016 (Presidencia de la República del Paraguay, 2016b; Presidencia de la República del Paraguay, 2016a). DINAVISA’s webpage makes available all forms required for the registry of biologics (DINAVISA, 2023).

Decree No. 6611/2016 categorizes requirements into two segments: general requirements (Table 3); and additional requirements (Table 4) for the registry of vaccines, hemoderivatives, innovative biologics, biosimilars, biologics for orphan diseases, and biologics obtained through recombinant DNA technology. Paraguay does not have specific regulation for biotechnological pharmaceutical products; their regulation is established within that of biologics.

**TABLE 3:** Paraguay’s general requirements for the sanitary registry of biologics for human use according to Decree No. 6611/2016 (Presidencia de la República del Paraguay, 2016a).

No.	Document	Description
1	Application forms	Approved by regent and legal representative.
2	RUE	Authenticated copy of valid unique company registration (RUE), granted by DINAVISA.
3	Free Sale Certificate	Authenticated copy of the certificate of free sale or documentation that accredits the commercialization of the drug issued by the Health Authority of the country of origin or provenance (as appropriate).
4	Manufacturer Approval	Authenticated copy of the approval of the manufacturer of the active ingredient, of the final product, and of the conditioner, issued by the competent health authority of the country of origin, in case it is not included in the Certificate of Good Manufacturing and Control Practices or in the Good Storage and Warehousing Practices.
5	Good Practices Certification	An authenticated copy of the Certificate of Good Manufacturing and Control Practices of the manufacturer of the active ingredient, of the manufacturer of the final product and of the conditioner, and of the storage issued by the sanitary authority of the country of origin or provenance (as applicable).
6	Production Description	A copy of the production process of the active ingredient up to the obtaining the final product with the description of said process and the complete set of data describing the manufacturing and control process, up to the final storage.
7	Storage Practices Certificate	An authenticated copy of the certificate of Good Storage Practices of the applicant and of the company in charge of the storage of the drug, issued by DINAVISA.
8	Third-Party Relations	An authenticated copy of the statement of the relationship with third parties involved in the processes to obtain the final product, from the production process to the final product, if applicable.
9	Contractual Documents	An authenticated copy of the contract of representation, or alternatively the contract of distribution, or contract of manufacturing, as applicable.
10	Risk Management Plan	Information requirements set on Annex III of Decree No. 6611/2016.
11	Technical-Scientific Data	Preclinical and clinical studies and any other information related to the development, quality, efficacy and safety of the product. In the case of biosimilar products, comparability studies with the innovator product. Also a stability study must be performed.
12	Foreign Document Authentication	All documents of foreign origin must be duly authenticated, consularized, or apostilled and legalized, and if written in a different language, accompanied by a translation into Spanish by a translator registered in the Supreme Court of Justice. Likewise, all documents must be valid on the date of the application.

**TABLE 4:** Paraguay’s specific additional requirements for the registry of vaccines, hemoderivatives, innovative biologics, biosimilars, biologics for orphan diseases, and biologics obtained through recombinant DNA technology.

Type of biologic	Specific requirements	Regulated by
Vaccines^a^	• Information about the active principle and the final product (requisites further explained in the regulation).	Annex I of Decree No. 6611/2016 (Presidencia de la República del Paraguay, 2016b)
	• Nonclinical study reports, which for *novel vaccines* encompass information on pharmacology, pharmacokinetics, toxicology (requisites further explained in the regulation), and an assessment of the possible shedding of the microorganism (for attenuated vaccines); while for *conventional vaccines*, they include bibliographic information supporting the pharmacodynamic or safety information for the formulation subject of the application for registration.	
	• Clinical study reports (Phases I, II and III).	
	The commercial release of each batch of imported vaccine must be requested separately through the application form approved by Resolution DINAVISA No. 64/2022.	
Hemoderivatives	• Applicants must submit to DINAVISA a “Plasma Master File”: documentation separate from the marketing authorization dossier containing detailed information on the characteristics of all human plasma used as starting/raw material for manufacturing subfractions, fractions, excipient components, and active ingredients. Each human plasma fractionation/processing center or establishment is responsible for preparing and keeping this information up-to-date within their Plasma Master File. The applicant or holder of the registry assumes responsibility for the drug.	Annex I of Decree No. 6611/2016 (Presidencia de la República del Paraguay, 2016b)
	• The Plasma Master File must: include information such as the origin, quality, and safety of the plasma; outline the system of collaboration between manufacturers or processing entities and collection/testing centers; specify the conditions of their interaction and specifications agreed among them, and list the medicinal products for which the file is valid.	
	• When submitting a complete dossier for the evaluation and certification of medicinal products not yet authorized, it is required to include a Plasma Master File. Applicants must provide all available preclinical and clinical information to demonstrate the safety and efficacy of the drug under evaluation compared to the reference biologic. Alternatively, if the drug is already registered with one of the specified Regulatory Health Agencies mentioned in article 4 of Decree No. 6611/2016, this information can also be provided. The Plasma Master File will undergo a scientific and technical evaluation by DINAVISA. A positive evaluation will result in the issuance of a certificate of compliance for the Plasma Master File along with an accompanying report. The file must be updated and recertified annually.	
Innovative biologics	• Quality studies: comprehensive information regarding the physicochemical, biological and immunological properties of both the active ingredient and the final product.	Decree No. 6611/2016, article 7 (Presidencia de la República del Paraguay, 2016a)
	• Efficacy, safety and immunogenicity studies: specific criteria for demonstrating effectiveness, safety and immune response will vary depending on the type of biological drug and will be determined on a case-by-case basis as specified in the annexes of the Decree following international guidelines set by WHO or ICH. The reports required include preclinical pharmacokinetics, pharmacodynamics, toxicity, immunogenicity, and interaction studies. For clinical evaluation, the reports must cover phase I trials, phase II trials, phase III studies, phase IV studies (if any), immunogenicity studies, and interaction studies.	
Biosimilar drugs	• These drugs must demonstrate the biosimilarity of the biological drug in terms of quality, through complete physicochemical and biological characterization by side-by-side comparison with the reference drug.	Decree No. 6611/2016, article 8 (Presidencia de la República del Paraguay, 2016a)
	• Preclinical and clinical studies needed to demonstrate biosimilarity regarding efficacy, safety and immunogenicity will follow specific requirements determined on a case-by-case basis, following WHO or ICH international guidelines. These aspects must align with guidelines for innovative biologics, laid out in article 7, paragraph B of Decree No. 6611/2016.	
	• The preclinical and clinical studies for the comparability exercise must be conducted with the same product applying for registration, in the same pharmaceutical form, concentration, dosage and route of administration as the reference drug; and must be multicenter, randomized, with a statistically significant number of patients as defined in the approved protocol, and in the same indications approved for the reference drug; conducted in centers authorized by the competent Health Authority where the studies are conducted.	
	• The reference drug shall be the innovator drug, except for proteins obtained through first-generation recombinant DNA technology (specified in article 3, paragraph b.1 of Decree No. 6611/2016) and pegylated proteins.	
	• The characterization of the product to be registered must be performed with appropriate techniques for the determination of physicochemical properties, biological activity, immunochemical properties and impurities. These criteria must be considered as key elements when planning the comparability exercise, taking into account the complexity of the molecular entity involved; and depending on the physicochemical properties of the molecule, the battery of tests must be extended.	
	• Comparative preclinical and clinical studies must be performed. The extent of this study will depend on the difference obtained with the innovator during the analysis. The requirements for conducting preclinical and clinical studies, their depth, and breadth will be determined by the nature and structural complexity of the active substance, information on the clinical behavior of the biological drug (including immunogenicity), and impurities profile.	
	• The biosimilar drug must be registered or approved with one of the Regulatory Agencies of the countries referred to in article 4 of Decree No. 6611/2016, paragraphs a, c, and d.	
	• Biologics obtained through manufacturing processes clearly different from the ones used for the reference drug will be evaluated on a case-by-case basis according to the nature of the drug.	Decree No. 6611/2016, article 9 (Presidencia de la República del Paraguay, 2016a)
	For monoclonal antibodies, in addition to the requirements previously mentioned for biosimilars, detailed information must be presented on:	Decree No. 6611/2016, article 10 (Presidencia de la República del Paraguay, 2016a)
	• Starting materials (cell lines).	
	• Production.	
	• Active ingredient.	
	• Preclinical and clinical trials to an extent consistent with the nature of the product and therapeutic indication, and evidence of similarity found during the comparability exercise in the characterization phases of physicochemical properties, biological activity and impurities.	
	Depending on the information provided, it will be determined whether additional preclinical, clinical, and quality information is required.	
	The extrapolation of indications will be evaluated case by case.	
Biologics for orphan diseases	• Application for importation, signed by the treating physician, stating the patient’s name, specifying the orphan disease and the quantity of medicines to be imported, with the period of time in which they will be used.	Annex VI of Decree No. 6611/2016 (Presidencia de la República del Paraguay, 2016b)
	• Original prescription and its copy, stating the patient’s name, name of the drug and active ingredients, total amount of the drug for a treatment that cannot exceed 60 days.	
	• Informed consent signed by the patient and the treating physician.	
	• Medical diagnosis and summary of medical history.	
	• All the aforementioned documents must be signed and stamped by the treating physician and they will have the nature of a sworn statement, the treating physician being exclusively responsible for the use of the biological drug on the patient.	
	• Copy of the patient’s identification document.	
	• Copy of the treating physician’s identification document and current professional registration.	
	• Copy of the prospectus or quali-quantitative formula of the active ingredients of the product to be imported or reliable scientific documentation that supports the use of the medicinal specialty.	
	• Copy of the remittance or invoice of origin, where the batch and expiration date of the biological medicine is stated once it has entered the country.	
Biologics obtained through recombinant DNA technology	• Information regarding the quality of the drug.	Scope defined: Decree No. 6611/2016, article 3, paragraph b.1 (Presidencia de la República del Paraguay, 2016a)
	• Certificate of free sale.	Specific requirements: Decree No. 6611/2016, article 12 (Presidencia de la República del Paraguay, 2016a)

^a^ Conventional vaccines are those that already meet the requirements of the WHO, or monographs in international pharmacopoeias, or are part of the immunization programs included in internationally recommended schedules; while novel vaccines are those for which there is no history of safety and efficacy, either because there is no known vaccine against the microorganism to be prevented, or because they contain a new combination of antigens, a new pharmaceutical form, a new route of administration, new adjuvants, or new preservatives (Presidencia de la República del Paraguay, 2016a).

The registration of all products requires preclinical and clinical trial information, and in the case of biosimilars, said studies must be done in a comparative manner. Paraguay does not require the performance of local clinical trials. The biosimilarity study seeks to rationally predict the same safety and clinical efficiency between the innovator product and the biosimilar. The process must define the quality and safety attributes necessary for comparison and must include preclinical and clinical studies. The scope of this study shall be defined based on the characteristics of the product and the differences in the production and purification mechanisms, and the results obtained may determine the need for additional studies for biosimilarity testing. The World Health Organization (WHO) has recently issued a new guideline on biosimilars (Kurki et al., 2022; WHO, 2022), thus we can expect local regulation instruments to be updated soon.

Vaccines present different situations. In the case of novel vaccines, preclinical and clinical studies are required; while for conventional and combined vaccines developed by new manufacturers, the demonstration of non-inferiority with marketed vaccines of proven efficacy and safety is required. When new vaccination schedules or new indications are proposed, they must be accompanied by the corresponding clinical trials.

Good Manufacturing and Control Practices are required in all cases. The guidelines for the certification of compliance of Good Manufacturing and Control Practices were approved through MSPyBS Resolution No. 020/2015 (MSPyBS, 2015a; MSPyBS, 2015b).

Sanitary registries are valid for 5 years, and can be renewed for similar periods. The renewal of sanitary registries for biologics necessitates compliance with the requirements specified in article 6 of the Decree, ensuring that there have been no changes in the production process from the active ingredient to the final product, modifications of the therapeutic indications, change of manufacturer, and other changes that DINAVISA considers essential to maintain the quality, safety, efficacy and immunogenicity of the biologic^2^. If there have been changes in the production process from the active ingredient to the final product, modifications of the therapeutic indications, change of manufacturer and other changes that DINAVISA considers essential to maintain the quality, safety, efficacy and immunogenicity of the biologic, a new application for sanitary registry must be submitted.

Regarding pharmaceutical products in Mercosur, several important resolutions have been incorporated into the Paraguayan regulatory framework. These include standards for registering pharmaceutical products across member states (Mercosur GMC, 1995; Presidencia de la República del Paraguay, 1997), criteria for company registration (Mercosur GMC, 1996c; Presidencia de la República del Paraguay, 1997), and detailed requirements for documentation and information necessary for registration (Mercosur GMC, 1996d; Presidencia de la República del Paraguay, 1997). In addition, standards for good manufacturing practices have been established to ensure product quality and safety (Mercosur GMC, 2009; Presidencia de la República del Paraguay, 2012b).

## 7 Biologics for veterinary use

Paraguay’s regulation of veterinary products, supervised by SENACSA, is characterized by a multilayered approach aimed at ensuring the safety and efficacy of veterinary biologics. The foundational legal framework, established by Law No. 667/1995 (República del Paraguay, 1995) and amended by Law No. 2426/2004 (República del Paraguay, 2004a), mandates the registration of all veterinary products and entities dealing with such products.

Meanwhile, Decree No. 6991/2017 (Presidencia de la República del Paraguay, 2017) entrusts SENACSA with exclusive jurisdiction over the authorization, operation, and supervision of entities dealing with veterinary products. The registration of all activities related to veterinary products is managed through SENACSA’s SIGOR (Regional Office Management Information System) online platform (SENACSA, 2021b; SENACSA, 2021c).

Resolution SENACSA No. 2803/2011 (SENACSA, 2011) outlines the criteria for the registration and licensing of importers. In addition, Resolution SENACSA No. 199/2012 (SENACSA, 2012a; SENACSA, 2018a) sets operational standards for vaccine-vending houses and distribution centers, while Resolution SENACSA No. 785/2012 (SENACSA, 2012b) sets comprehensive guidelines covering a wide array of operational aspects of laboratories including personnel management, facility layout, and quality control processes.

Biosafety information requirements for the registration of veterinary biologics include biological and chemical composition, specifications and methods of control for components of the formula and for culture media, substrates and other biological materials used, methodology of product manufacturing, method of control of the finished product, and evidence of safety and efficacy (literature review and clinical trials, when applicable) (SENACSA, 2020; SENACSA, 2021b).

The registration process for subunit immunogens produced via biotechnological processes mandates comprehensive control of the components and final product, ensuring their safety, quality, and efficacy. This encompasses chemical and biological characterization, manufacturing processes, and quality control protocols. The registration process focuses specifically on managing biological risks, emphasizing the prevention of public, animal, and environmental health hazards during production (SENACSA, 2021b; SENACSA, 2021a).

Disease control is another critical aspect addressed. Resolutions SENACSA No. 687/2017, No. 1641/2017, and No. 124/2018 focus on foot-and-mouth disease vaccine production standards, while Resolution SENACSA No. 690/2017 outlines standards for *Brucella abortus* vaccine production. These standards include infrastructure requirements for the manufacturers, dosage and specific strains to be used in production, and quality control (SENACSA, 2017b; SENACSA, 2017c; SENACSA, 2017a; SENACSA, 2018b).

Mercosur GMC Resolutions scaffold the harmonization of regional regulatory frameworks for veterinary products. In particular, vaccines against diseases such as symptomatic anthrax and gas gangrene are addressed, along with vaccine production in poultry. The adoption of Mercosur resolutions into Paraguay’s regulatory framework indicates an effort to align veterinary health standards and promote a unified strategy for animal healthcare (Table 5).

**TABLE 5:** Resolutions dealing with veterinary products approved by the Mercosur Common Market Group and incorporated into the Paraguayan regulatory framework.

Resolution GMC no.	Title	Incorporated to paraguayan regulatory framework through
11/93 (Mercosur GMC, 1993)	Regulatory framework for veterinary products	Presidential Decree No. 891/98 (Presidencia de la República del Paraguay, 1998b)
39/96 (Mercosur GMC, 1996a)	Supplementary regulations to the regulatory framework for veterinary products	Presidential Decree No. 891/98 (Presidencia de la República del Paraguay, 1998b)
40/96 (Mercosur GMC, 1996b)	Regulation of the validation system for veterinary products	Presidential Decree No. 891/98 (Presidencia de la República del Paraguay, 1998b)
77/96 (Mercosur GMC, 1996e)	Technical regulation for control of vaccines against symptomatic anthrax, gas gangrene, enterotoxemia, and tetanus	Presidential Decree No. 891/98 (Presidencia de la República del Paraguay, 1998b)
4/97 (Mercosur GMC, 1997)	Technical regulations for the production and control of vaccines, antigens, and diluents for poultry farming	Presidential Decree No. 891/98 (Presidencia de la República del Paraguay, 1998b)
33/00 (Mercosur GMC, 2000)	Mutual recognition agreements on genealogical records and animal genetic evaluations	Incorporation instrument unavailable/not found

## 8 Intellectual property of products of biotechnology

Patents are regulated in Paraguay through Law No. 1630/2000 (República del Paraguay, 2000), which establishes the requirements for the obtention of a patent, types of patent, matters excluded from patent protection, duration of the patent, and other relevant regulations. In particular, article 5 states that plants and animals (except microorganisms), and processes that are essentially biological for the production of plants or animals, are excluded from patent protection.

In addition, article 16 determines that during the application for a patent, when the invention refers to a product or procedure related to some biological material that is not available to the public and cannot be described in such a way that the invention can be implemented by a person skilled in the matter, the description shall be complemented by the deposit of said material in a deposit institution recognized by the General Directorate of Industrial Property. Such deposit shall not be required if it has already been made in any state member of the World Trade Organization or if the examination of novelty has already been carried out by the authority of any such country. The executing body of intellectual property policy is the National Directorate of Intellectual Property (República del Paraguay, 2012).

In compliance with article 27 section 3.b of Law No. 444/1994 (incorporation of TRIPS agreement) (República del Paraguay, 1994b), Law No. 385/1994 (República del Paraguay, 1994c) establishes several instruments for the protection of plant varieties, which apply to GM crops. Its implementing authority is SENAVE since the passing of Law No. 2459/2004 (República del Paraguay, 2004b). Further specifications on the use of these instruments can be found in Decree No. 7797/2000 (Presidencia de la República del Paraguay, 2000).

## 9 Difficulties with data collection

One of the primary difficulties in Paraguay’s regulatory framework for biotechnology is the absence of a centralized database of regulations. Currently, these regulations are scattered across various platforms, which often leads to accessibility issues, and some are entirely unavailable online, meaning interested parties must make a written request of a physical copy at the respective government office, resulting in extended waiting periods.

This decentralization impedes the ability to monitor the development of regulations, thereby complicating the process of determining whether a norm is currently in effect, or has been repealed, replaced, or modified. Paraguay has a unified online portal of public information (Portal Paraguay - Acceso a la Información Pública, 2023) where such requests can also be made, but they are not always answered in a timely manner. Additionally, the absence of official signatures or letterheads on digitally available documents necessitates additional verification steps, which further diminishes the efficiency of the system. We gathered the regulations analyzed for this work and deposited them in a repository to ensure their availability to our readers (in Spanish) (Benitez Candia et al., 2024).

Mercosur GMC Resolutions present a specific challenge. We were unable to find a local incorporation instrument for Resolution GMC No. 33/00 (Table 5), which results in uncertainty about its domestic status.

## 10 Considerations on the situation of the regulatory framework for biotechnology in Paraguay

The biotechnology framework in Paraguay is closely aligned with Mercosur, yet the regulatory agencies of the member states exhibit distinct characteristics that may result in variations in their approaches (Magnuson et al., 2013; Mukherjee et al., 2022). Cooperation within the region is primarily sustained by the exchange of information and a certain level of harmonization of legal and regulatory requirements. However, effective harmonization necessitates the acceptance of common values and objectives, shared interests and challenges, mutual economic and other advantages, avoidance of disputes, collaboration on other concerns, and streamlining of procedures (McLean et al., 2002). Unfortunately, achieving this level of harmonization is a daunting task.

Moreover, regulatory systems have often been implemented on a “piece-by-piece” basis (McLean et al., 2002) in response to the urgent needs of the moment, and are more reactive than preventive systems. An inventory and evaluation of priorities, policies, existing regulatory regimes, and scientific and technical means is ideally a prerequisite to the development and implementation of policies and regulations (McLean et al., 2002; Schoemaker et al., 2020). However, building such a system and making it operational is complicated by the fact that there is no single best approach nor standard that reflects cultural, political, financial, and scientific heterogeneity. When establishing a regulatory framework, considerable attention must be paid to factors such as regulatory triggers, transparency, public involvement in policy-making and regulatory decision-making processes, and proportionate methods for assessing and managing risk.

While Paraguay has made efforts to improve its regulatory framework, the triggers for regulatory review are not adequately defined in several current norms. The country has recently implemented science-based approaches to assessing and managing risks in relation to GMOs. However, there is a deficiency in terms of public consultations and participation. Current processes for public engagement are lacking, resulting in a disconnect between regulatory bodies and the broader community. Establishing an effective public consultation process would not only enhance regulatory decision-making, but also promote a more transparent approach to biotechnology governance. It is essential to involve stakeholders and the public in discussions about biotechnology to ensure informed policymaking and foster trust.

Several regulations have proven particularly challenging in their interpretation. An example is article 10 of Law No. 3283/2007 (República del Paraguay, 2007b), which addresses the validation of evaluations for sanitary registration from specific countries. The wording of the law presents a challenge to understanding whether the procedure involves automatic acceptance from third-country assessments, and the extent of this provision. At the very least, interpretations are manifold. In addition, articles 4 and 5 of Decree No. 6611/2016 determine that for biologics with a sanitary registry from the regulatory agencies specified in the aforementioned law, therapeutic indications “can be recognized and expanded”, which makes interpretation of both regulations together even more challenging.

These difficulties have significant ramifications for a range of stakeholders, including researchers, industry experts, and policymakers; and can impede the development, authorization, and commercialization of biotechnology products, consequently affecting scientific progress. At minimum, the process and criteria for risk assessment and risk management must be widely published to instill trust in the system as credible and predictable among developers, stakeholders, and the public (Crow et al., 2016; Wolt and Wolf, 2018).

We suggest the evaluation of current scientific and technical capacity in Paraguay in order to facilitate the development of a more fit-for-purpose system. A sound regulatory system necessitates continuous updates on the latest scientific advancements; without such updates, the regulator’s knowledge base will have a limited lifespan. We hope that this initial assessment of current legislation will be the starting point for determining and implementing appropriate, scientifically sound regulations.

## Footnotes

1 Defined in Decree No. 6611/2016 as: “substances composed of or derived from proteins, nucleic acids, sugars or a complex combination of the above or living entities such as cells or tissues, obtained from living organisms or their tissues. They include viruses, therapeutic serums, toxins, antitoxins, vaccines, blood, blood components or derivatives, allergenic products, hormones, colony-stimulating factors, cytokines, antibodies, heparins, among others. The sources and methods of production include, but are not limited to cell culture, microorganisms, extraction from tissues or biological fluids, recombinant DNA techniques, transgenesis, hybridoma techniques, propagation of microorganisms in embryos or animals, etc. They are products used for the prevention, treatment, or *in vivo* diagnosis of certain diseases.”

2 Except for products specified in article 3, paragraph b.1 of the Decree, which must follow requirements established in article 12, specified in the section for biologics obtained through recombinant DNA technology (Table 4).
