# Supplementary material for: Paraguay’s approach to biotechnology governance: a comprehensive guide
Source: Front Bioeng Biotechnol. 2024 Mar 27;12:1373473. doi: 10.3389/fbioe.2024.1373473 (PMC11004369; doi:10.3389/fbioe.2024.1373473)
Supplement: Supplementary file 1 [file DataSheet1.docx]

Supplementary Material

# Supplementary Tables

Supplementary Table 1. Procedure for the risk management of activities with regulated GMOs in the agricultural sector Resolution SENAVE No. 283/2014 (SENAVE, 2014).

| Activities | Responsible parties | Procedures |
| --- | --- | --- |
| Normative responsibility | Technical General Directorate:  Directorate of Agricultural Biosafety  Directorate of Operations  Coordination of Regional Offices | - Oversight of risk management of GMO authorized by MAG |
| Document inspection | Importer | - Present chain of custody of GMO to be imported during importing process |
| Physical inspection | Phytosanitary inspector | - Check good condition of packaging and sealing of GMO, transport safety conditions from entry to destination in order to prevent accidental leak of GMO and that it will not be exposed to environment during transit |
| Storage | Phytosanitary inspector | - Verify that GMO propagation materials be stored in adequate conditions (secure, closed, and dry) before and after use - Know identity of the person responsible for safekeeping and storage of material - Submit report to Directorate of Agricultural Biosafety |
| Seed treatment | SENAVE inspector | - Oversee package unsealing and treatment of GM seeds - Verify that person(s) responsible for GM seeds provide adequate measures to prevent accidental leak outside seed treatment area - Verify GM seed resealing - In case of spillage, verify application of corrective measures - Keep record of seed treatment procedure |
| Supervision of regulated field trials (seeding, reseeding, crop development, border management, final disposal) | SENAVE inspector | - Verify distances to other crops or production or experimental plots at the time of sowing as indicated in the corresponding mag resolution - Verify absence of persons not involved in the procedure and animals in the trial area - Supervise sowing of boundary crops in the amount of rows indicated in the corresponding protocol - Ensure that there are no accidental leaks of the regulated GM material during entry and exit of authorized personnel, machinery and implements - Verify thorough cleaning of implements before removal from the plot - Verify resealing of remaining propagation materials - Ensure that thinned plants be left at trial site for decomposition - Ensure physical and temporal isolation of trial from other plots of the same species and relatives during the flowering period - Verify conditions of border rows - Supervise removal of reproductive material and destruction of plants from border rows. - Monitor harvesting of regulated material for final disposal, and activities carried out with harvest products (shelling, weighing, etc.) |

Supplementary Table 2. Resolutions dealing with animal genetic materials approved by the Mercosur Common Market Group and incorporated into the Paraguayan regulatory framework.

| Resolution GMC No. | Title | Incorporated to Paraguayan regulatory framework through |
| --- | --- | --- |
| 73/99 (Mercosur GMC, 1999) | Requirements and List of Mercosur Reference Laboratories and Alternative Laboratories for the Diagnosis of Animal Diseases | Decree No. 10846/2000 (Presidencia de la República del Paraguay, 2000) |
| 49/14 (Mercosur GMC, 2014) | Animal health requirements for the importation of frozen bovine and bubaline semen | Resolution SENACSA No. 1585/2019 (SENACSA, 2019)^[[1]](#footnote-1)^ |
| 7/17 (Mercosur GMC, 2017a) | Animal health requirements for the importation of frozen porcine semen | Resolution SENACSA No. 1585/2019 (SENACSA, 2019) |
| 44/17 (Mercosur GMC, 2017b) | Animal health requirements for the importation of bovine and bubaline embryos collected in vivo and/or produced in vitro | Resolution SENACSA No. 1585/2019 (SENACSA, 2019) |
| 32/18 (Mercosur GMC, 2018a) | Animal health requirements for the importation of frozen equine semen | Resolution SENACSA No. 1585/2019 (SENACSA, 2019) |
| 42/18 (Mercosur GMC, 2018b) | Modification of the animal health requirements for the importation of frozen bovine and bubaline semen | Resolution SENACSA No. 1585/2019 (SENACSA, 2019) |
| 43/18 (Mercosur GMC, 2018c) | Modification of the animal health requirements for the importation of equine embryos | Resolution SENACSA No. 1585/2019 (SENACSA, 2019) |
| 15/19 (Mercosur GMC, 2019a) | Animal health requirements for the importation of caprine embryos collected in vivo | Resolution SENACSA No. 1585/2019 (SENACSA, 2019) |
| 16/19 (Mercosur GMC, 2019b) | Animal health requirements for the importation of ovine embryos collected in vivo | Resolution SENACSA No. 1585/2019 (SENACSA, 2019) |
| 18/19 (Mercosur GMC, 2019c) | Animal health requirements for the importation of frozen ovine semen | Resolution SENACSA No. 1585/2019 (SENACSA, 2019) |
| 19/19 (Mercosur GMC, 2019d) | Animal health requirements for the importation of frozen caprine semen | Resolution SENACSA No. 1585/2019 (SENACSA, 2019) |

# References

Mercosur GMC (1999). Resolución 73/99 Requisitos y Nómina de Laboratorios de Referencia y Laboratorios de Alternativa del MERCOSUR para Diagnóstico de Enfermedades Animales. Available at: https://normas.mercosur.int/public/normativas/1447 (Accessed November 3, 2023).

Mercosur GMC (2014). Resolución 49/14 Requisitos zoosanitarios de los estados partes para la importación de semen bovino y bubalino congelado (Derogación de la Res. GMC n° 32/14). Available at: https://normas.mercosur.int/public/normativas/3015 (Accessed November 3, 2023).

Mercosur GMC (2017a). Resolución 07/17 Requisitos zoosanitarios de los estados partes para la importación de semen porcino congelado. Available at: https://normas.mercosur.int/public/normativas/3369 (Accessed November 3, 2023).

Mercosur GMC (2017b). Resolución 44/17 Requisitos zoosanitarios de los estados partes para la importación de embriones bovinos y bubalinos colectados in vivo y/o producidos in vitro (Derogación de la Res. GMC n° 25/10). Available at: https://normas.mercosur.int/public/normativas/3508 (Accessed November 3, 2023).

Mercosur GMC (2018a). Resolución 32/18 Modificación de los requisitos zoosanitarios de los estados partes para la importación de semen equino congelado (Modificación de la Resolución GMC n^o^ 13/14). Available at: https://normas.mercosur.int/public/normativas/3597 (Accessed November 3, 2023).

Mercosur GMC (2018b). Resolución 42/18 Modificación de los requisitos zoosanitarios de los estados partes para la importación de semen bovino y bubalino congelado (Modificación de la Resolución GMC n° 49/14). Available at: https://normas.mercosur.int/public/normativas/3623 (Accessed November 3, 2023).

Mercosur GMC (2018c). Resolución 43/18 Modificación de los requisitos zoosanitarios de los estados partes para la importación de embriones equinos (Modificación de la Resolución GMC n^o^ 42/07). Available at: https://normas.mercosur.int/public/normativas/3624 (Accessed November 3, 2023).

Mercosur GMC (2019a). Resolución 15/19 Requisitos zoosanitarios de los estados partes para la importación de embriones caprinos recolectados in vivo (Derogación de la Resolución GMC n° 47/14). Available at: https://normas.mercosur.int/public/normativas/3728 (Accessed November 3, 2023).

Mercosur GMC (2019b). Resolución 16/19 Requisitos zoosanitarios de los estados partes para la importación de embriones ovinos recolectados in vivo (Derogación de la Resolución GMC n° 48/14). Available at: https://normas.mercosur.int/public/normativas/3729 (Accessed November 3, 2023).

Mercosur GMC (2019c). Resolución 18/19 Requisitos zoosanitarios de los Estados partes para la importación de semen ovino congelado (Derogación de las Resoluciones GMC n° 14/13 y 54/14). Available at: https://normas.mercosur.int/public/normativas/3731 (Accessed November 3, 2023).

Mercosur GMC (2019d). Resolución 19/19 Requisitos zoosanitarios de los estados partes para la importación de semen caprino congelado (Derogación de las Resoluciones GMC n° 15/13 y 55/14). Available at: https://normas.mercosur.int/public/normativas/3732 (Accessed November 3, 2023).

Presidencia de la República del Paraguay (2000). Decreto N^o^ 10846/2000 Por el cual se dispone la vigencia en la República del Paraguay de normativas Mercosur, referente a armonizaciones de medidas y requisitos fitosanitarios, semillas y zoosanitarios. Available at: https://doi.org/10.5281/zenodo.10688936 (Accessed December 28, 2023).

SENACSA (2019). Resolución N^o^ 1585/2019 Por la cual se dispone la vigencia en la República del Paraguay de las Resoluciones actualizadas y aprobadas por el Grupo Mercado Común del Mercosur, referente a armonizaciones de requisitos zoosanitarios. Available at: https://doi.org/10.5281/zenodo.10688936 (Accessed December 28, 2023).

SENAVE (2014). Resolución N^o^ 283/2014 Por la cual se aprueba el procedimiento para la gestión del riesgo en actividades con organismos genéticamente modificados regulados en el ámbito agrícola. Available at: http://web.senave.gov.py:8081/docs/resoluciones/senave/Res283-14.pdf (Accessed November 3, 2023).

1. Res. GMC No. 18/19 repeals Res. GMC No.54/14, and Res. GMC No.19/19 repeals Res. GMC No. 55/14. Res. SENACSA 1585/2019 incorporates all four mentioned GMC Resolutions into the Paraguayan regulatory framework. [↑](#footnote-ref-1)
